# Supplementary material for: Model‐free machine learning‐based 3D single molecule localisation microscopy
Source: J Microsc. 2025 May 8;299(1):77–87. doi: 10.1111/jmi.13420 (PMC12166345; doi:10.1111/jmi.13420)
Supplement: Supplementary file 1 — Supporting Information [file JMI-299-77-s002.pdf]

SUPPORTING INFORMATION

Figure 8 illustrates the location of emitters on the NUP96 nuclear pore complex.

Figure 9 presents three "successful" nuclear pore reconstructions for each Z localisation methodology (*easyZloc*, *DECODE*, *FD-DeepLoc* along with corresponding reconstructions of the same data using the other methodologies.

Supplementary Video 1 presents a comparison of reconstructions of all the nuclear pores which were "successfully" reconstructed by at least one Z localisation methodology.

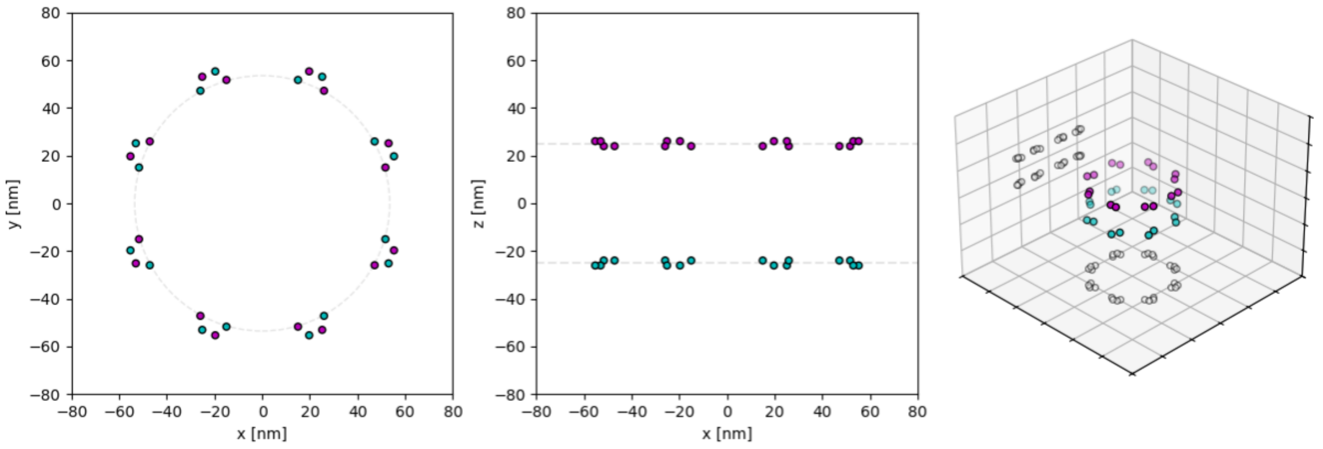

FIGURE 8: Schematic of NUP96p nuclear pore proteins viewed from top and side and as a 3D plot. The separation of the rings is 46 nm[38].

EasyZloc

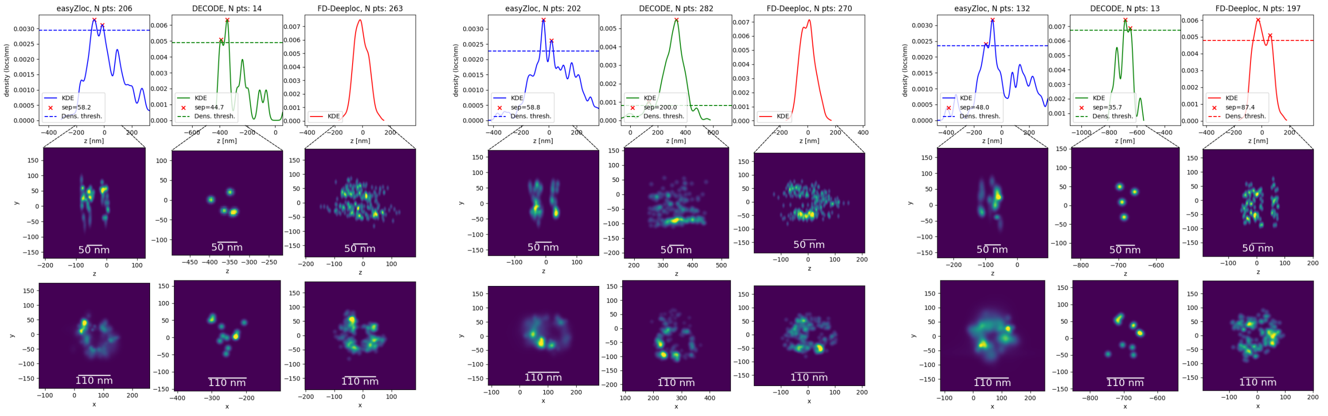

(a) Selected examples of nuclear pores which were "successfully" reconstructed using *easyZloc*.

DECODE

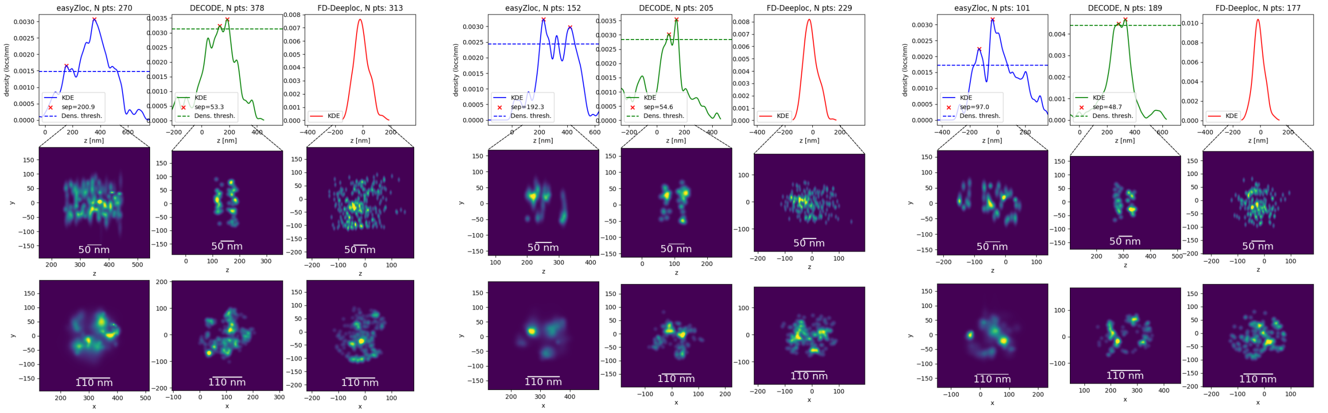

(b) Selected examples of nuclear pores that were "successfully" reconstructed using *DECODE*.

FD-DeepLoc

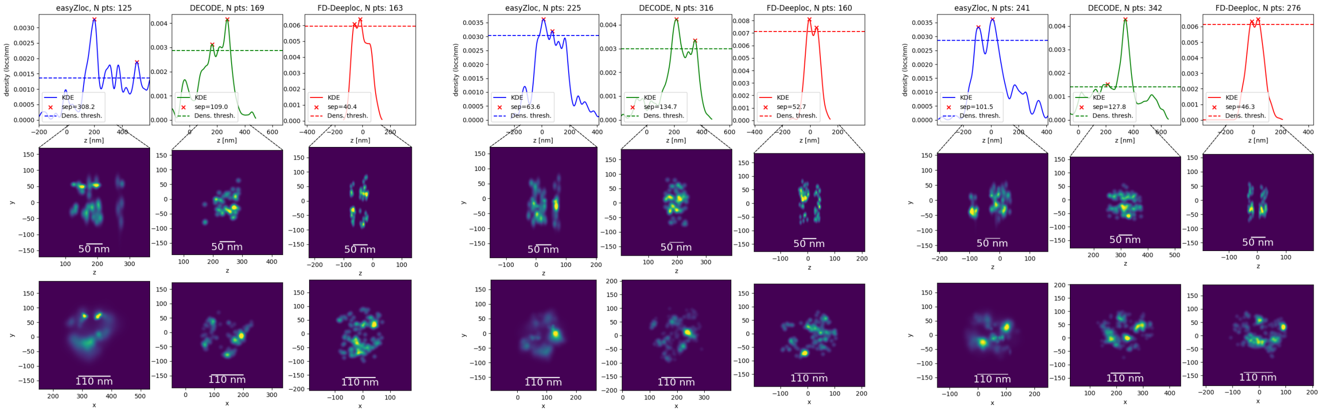

(c) Selected examples of nuclear pores that were "successfully" reconstructed using *FD-DeepLoc*.

FIGURE 9: Additional examples of nuclear pore reconstructions
